# Psychometric validation of the Hand Disability in Systemic Sclerosis-Digital Ulcers (HDISS-DU®) patient-reported outcome instrument

**DOI:** 10.1186/s13075-019-2087-4

**Published:** 2020-01-06

**Authors:** Luc Mouthon, Serge Poiraudeau, Margaret Vernon, Kelly Papadakis, Loïc Perchenet, Dinesh Khanna

**Affiliations:** 1 Department of Internal Medicine, Reference Center for Rare Systemic Autoimmune Diseases of Ile de France, Hôpital Cochin, Assistance Publique-Hôpitaux de Paris, Université Paris Descartes, Sorbonne Paris Cité, Paris, France; 2 Department of Rehabilitation, Hôpital Cochin, Assistance Publique-Hôpitaux de Paris, Université Paris Descartes, Sorbonne Paris Cité, Paris, France; 3 Evidera, Bethesda, MD USA; 4 Actelion Pharmaceuticals Ltd., Allschwil, Switzerland; 5 Department of Internal Medicine, University of Michigan Scleroderma Program, Suite 7C27, 300 North Ingalls Street, Ann Arbor, MI USA

**Keywords:** Patient perspective, Qualitative research, Systemic sclerosis, Treatment

## Abstract

**Background:**

We aimed to develop a patient-reported outcome measure, in accordance with the US Food and Drug Administration guidance, to capture the impact of systemic sclerosis-related digital ulcers (SSc-DUs) on hand function. Psychometric analyses were conducted to evaluate and document the measurement properties of the resulting instrument—the Hand Disability in Systemic Sclerosis-Digital Ulcers (HDISS-DU®).

**Methods:**

The HDISS-DU was developed through a series of confirmatory, qualitative concept-elicitation interviews (*N* = 36) to provide supportive evidence that the instrument captures all relevant issues and functional limitations relating to SSc-DUs in this patient population. Psychometric analyses used blinded data from two randomised, controlled, phase 3 trials in patients with SSc-DUs (*N* = 517). The analyses included assessment of reliability, construct validity, responsiveness and thresholds for meaningful change.

**Results:**

Qualitative interviews confirmed that the HDISS-DU had good content coverage and patients understood the HDISS-DU instructions, items and response scale. The HDISS-DU demonstrated excellent internal consistency and test-retest reliability, with satisfactory construct validity. Overall, the HDISS-DU was highly responsive to change in digital ulcer severity: the no-change group (for other criterion measures) had mean differences and effect sizes close to 0, while mean differences were mostly negative (indicating improvement) for the improvement groups (for other criterion measures) and vice versa. The preliminary threshold for meaningful change was a 0.50 difference in HDISS-DU score.

**Conclusions:**

Using data from two large studies of SSc-DU patients, these psychometric analyses support the reliability, validity, discriminating ability and responsiveness to change of the HDISS-DU for evaluating treatment outcomes in future clinical studies and clinical practice.

## Background

Digital ulcers (DUs) are one of the most frequent and burdensome clinical manifestations of progressive vascular disease in systemic sclerosis (SSc) [1]. Approximately half (44–60%) of patients with SSc will experience at least one DU [2], with many suffering non-healing or recurrent DUs, refractory to intervention. These painful skin lesions, areas of denuded tissue affecting the dermal and epidermal skin layers [3], most frequently affect the fingertips and may involve several fingers simultaneously. DUs severely limit patients’ everyday tasks, causing severe functional disability and significantly impacting quality of life [4–6]. Patients with SSc-related DUs (SSc-DUs) can experience anxiety, associated social issues and self-image problems [6]. Furthermore, the soft tissue and/or the underlying bone frequently becomes infected, potentially leading to gangrene and amputation, without appropriate intervention [7].

Currently, the most frequently used measures of change in DU status in trials are clinical assessments of overall DU count and presence/absence of new DU(s) since the last assessment (e.g. [8]). However, these endpoints do not encompass the full spectrum of symptoms such as pain, impaired hand function and disability, or incorporate patients’ perspectives in the evaluation of treatment response. Patient-reported outcomes (PROs) provide valuable and unique information on the impact of a medical condition and the effectiveness of an intervention from a patient’s perspective; therefore, they can be utilised to ensure a comprehensive assessment of an intervention [9]. Patient-reported outcome measures (PROMs) that are intended for use as primary or key secondary endpoints in clinical trials should be developed and psychometrically evaluated in accordance with the US Food and Drug Administration’s (FDA) guidance [10]. To our knowledge, there are no existing PROMs for SSc-DUs that meet FDA standards. Specifically, there is no published evidence that current measures were developed with patient input to capture all clinically relevant issues and limitations that are meaningful to patients with the disease [10], nor is there evidence that measures are easily understood by patients (as intended), and have been psychometrically validated in this population to document their measurement properties (reliability, content validity and sensitivity to change) [10].

The Cochin Hand Function Scale (CHFS) is an existing PROM to assess hand disability in SSc-DUs, originally developed for rheumatoid arthritis [11]. It is a self-administered, 18-item questionnaire about activities related to daily life that is reliable and has good construct validity in SSc [12, 13]. Patients with SSc-DUs did not provide input during the CHFS development, and content validity and other measurement properties of the scale have not yet been established in this patient population.

The objectives of the present research were to adapt the CHFS, in accordance with FDA guidance, to capture the patient-reported impact of DUs on hand function in patients with SSc and to evaluate the psychometric properties of the resulting instrument—the Hand Disability in Systemic Sclerosis-Digital Ulcers (HDISS-DU®).

## Methods

**Fig. 1:**
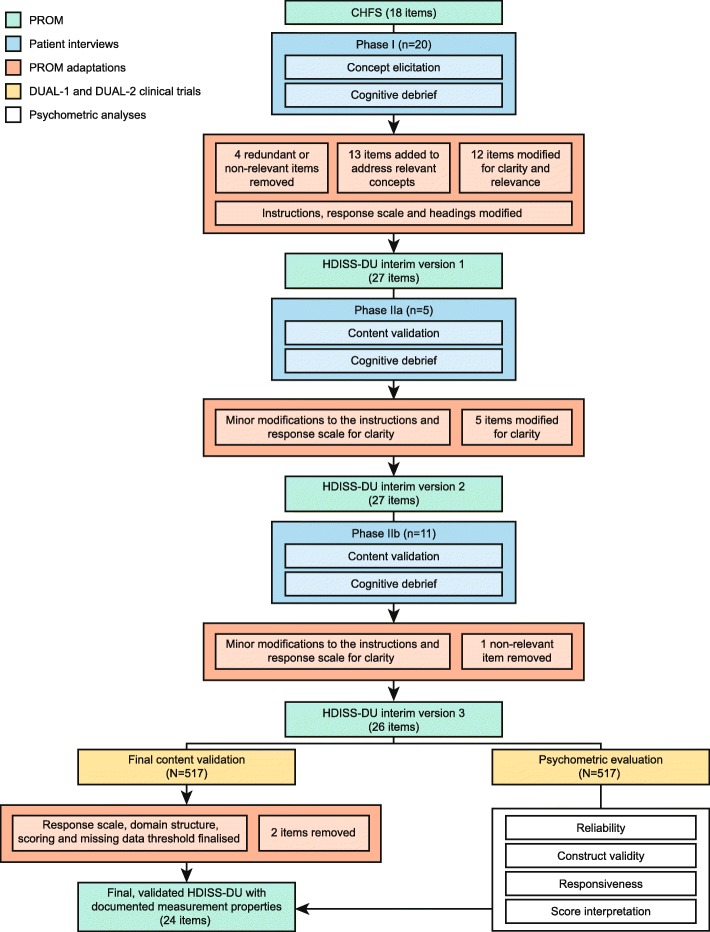
Schematic of the development, content validation and psychometric evaluation of the HDISS-DU instrument. CHFS, Cochin Hand Function Scale; HDDIS-DU, Hand Disability in Systemic Sclerosis-Digital Ulcers; n, number of participants; PROM, patient-reported outcome measure; SSc-DU, systemic sclerosis-related digital ulcer

The development and psychometric evaluation of the HDISS-DU are summarised in Fig. 1.

### Development and content validity

A cross-sectional, qualitative research study was undertaken to adapt the CHFS to capture the impact of DUs on hand function in patients with SSc-DUs and assess the content validity of the resulting instrument—the HDISS-DU—in this patient population. Participant interviews were conducted in two phases: concept elicitation and cognitive debrief for the CHFS (phase I) and content validation and cognitive debrief for the HDISS-DU (phase II). One-to-one interviews were conducted by experienced interviewers face-to-face or by telephone, using study-specific interview guides. Interviews were audio-recorded, transcribed by a third-party transcription agency and qualitatively analysed using ATLAS.ti v5.0.

#### Participants

Participants were recruited at five clinical sites across the USA through advertisements posted on SSc patient websites. Eligible participants were ≥ 18 years of age, with a diagnosis of SSc (according to the 1980 American College of Rheumatology [ACR] criteria) [14], and ≥ 1 visible, active, ischemic DU located (i) on the palmar surface, (ii) at or distal to the proximal interphalangeal joints or (iii) at the digital tip, for which the patient had seen a physician within the past 8 weeks. As this study was conducted prior to 2013, the ACR/European League Against Rheumatism criteria [15] were not used. Major exclusion criteria are detailed in Additional file 1: Method S1.

#### Qualitative patient interviews

Firstly, open-ended questions collected qualitative data on patients’ experiences with SSc-DUs and functional limitations affecting daily life activities (concept elicitation). These data ensured that the adapted PROM covered all issues and limitations relevant for this patient population. Participants then self-administered the CHFS [11], and interviewer-led cognitive debriefing assessed the participants’ understanding of the CHFS items and ease of self-administration. Data collected during the phase I interviews were used to adapt the CHFS and develop a new instrument—the HDISS-DU.

To confirm the changes to the CHFS and assess the content validity of the new instrument, the second phase of interviews was conducted. Interviews and subsequent modification to interim versions of the HDISS-DU were performed iteratively until cognitive debriefing suggested that the HDISS-DU provided a comprehensive measure of functional limitations relating to SSc-DUs and that each item was relevant and clear.

At each interview, participants completed a single-item global pain scale (self-assessment of pain at its worst) and two patient global assessment questions (assessing DU severity and global change in illness severity). Following each interview, participants completed a Scleroderma Health Assessment Questionnaire (SHAQ) assessing self-reported function [16].

### Psychometric validation study

#### Study design

Data from two randomised, placebo-controlled, double-blind, multicentre, parallel-group, phase 3 trials were used to assess the psychometric properties of the HDISS-DU. Both trials evaluated the efficacy of the tissue-targeting dual endothelin receptor antagonist—macitentan—in reducing the number of new DUs in patients with SSc. The methodology and results of DUAL-1 (NCT01474109) and DUAL-2 (NCT01474122) have been published previously [17]. In brief, they included 289 and 265 adult patients with SSc and ≥ 1 visible active ischemic DU, randomised to either 3 mg macitentan, 10 mg macitentan or placebo. The HDISS-DU was self-administered by patients at baseline and weeks 4, 8, 12 and 16.

Final content validation and the psychometric analyses were performed on a blinded, pooled dataset of all randomised patients with complete baseline information in DUAL-1 and DUAL-2 (*N* = 517; Fig. 1). Analyses were conducted using SAS/STAT® software (SAS Institute Inc., Cary, NC, USA). A *p* value of < 0.05 was used to assess the significance of statistical tests. Acceptable thresholds in these analyses included intraclass correlation coefficient (ICC) ≥ 0.80 [18], Cronbach alpha > 0.70 [18], Spearman’s rho (*r*) ≥ 0.40 (convergent validity) or ≤ 0.40 (discriminant validity) [19], and small (≥ 0.20), moderate (≥ 0.50) or large effect size (≥ 0.80) [20].

#### Final content validation

HDISS-DU responses were summarised descriptively, and the potential for differential item functioning was investigated (Additional file 1: Method S2). The domain structure was determined using exploratory factor analysis with oblique rotation methods and data from baseline and week 8 (and week 16 if large differences between the results at these time points). Eigenvalues (minimum criterion < 1) and factor loadings were reviewed in order to identify the final factor structure, looking for a simple structure with meaningful interpretation and factor loadings > 0.4 [21].

Item redundancy was explored using inter-item Spearman’s rank-order correlation, with highly correlated item pairs (*r* > 0.80) flagged for potential removal and investigated further for test-retest reliability, responsiveness to change and effect size (Additional file 1: Method S2). Rasch modelling was undertaken to identify items that did not fit the scoring assumptions (i.e. expected patient-specific severity) [22]. Confirmatory factor analysis assessed the stability of the final domain structure over time [21], comparing responses at baseline and week 8 (and week 16 if large differences between the results at these time points). Consecutive item removal and mean score stability (calculated from non-missing items) confirmed the suitability of a mean overall score and defined a missing data threshold.

#### Evaluation and measurement of psychometric properties

A summary of the HDISS-DU and other measures assessed in DUAL-1 and DUAL-2 and utilised in the present psychometric analyses is presented in Additional file 1: Table S3.

##### Reliability

Internal consistency reliability was measured using Cronbach’s alpha and ‘Alpha if item deleted’ analyses established whether item removal would improve internal consistency. Test-retest reliability was assessed by evaluating the HDISS-DU score reproducibility at baseline and week 4 in stable patients (defined by the scores from other criterion measures) using ICCs and paired *t* tests. For these analyses, there were four definitions of stable patient groups: ‘no change’ at week 4 for (1) patient-reported or (2) physician-reported global change, and identical scores at baseline and week 4 for (3) patient-reported or (4) physician-reported severity.

##### Construct validity

Construct validity was assessed using Spearman’s rank-order correlation and a priori hypotheses on instruments assessed in DUAL-1 and DUAL-2 that capture similar concepts (convergent validity) and those that capture different concepts (discriminant validity) to the HDISS-DU. Correlations were defined as strong (*r* ≥ 0.50), moderate (*r* ≥ 0.30 to < 0.50) or weak (*r* < 0.30) [19]. Known-group validity was assessed by either independent samples *t* test (two patient groups) or by analysis of variance (≥ 3 patient groups) for patients grouped by either number of DUs (≤ 3, > 3), DU complications category (none, mild, moderate, severe) or number of hands affected (one, two).

##### Responsiveness

HDISS-DU score responsiveness was assessed by analysis of covariance, and effect size was calculated following the methodology described in Additional file 1: Method S2. Responsiveness was further investigated by descriptive statistics for the 16-week change in HDISS-DU score by the cumulative number of new DUs in this time.

##### Minimal meaningful change threshold

An anchor-based approach was utilised to identify the minimal meaningful change in HDISS-DU score, as this may help define responders in a clinical trial setting. The 16-week change in HDISS-DU score was calculated for four patient groups who exhibited minimal meaningful change (defined by the scores on other criterion measures): an improvement of one category at week 16 for (1) patient-reported or (2) physician-reported severity, and ‘minimally improved’ or ‘minimally worse’ at week 16 for (3) patient-reported or (4) physician-reported global change.

### Patient and public involvement

Patients provided input into the HDISS-DU developed through participation in qualitative research interviews and content validation. This ensured the resulting instrument covered all relevant issues and functional limitations relating to this patient population and that each item was easily understood, with an appropriate response scale and recall period from patients’ perspectives.

## Results

### Development and content validity

Interviews took place from December 2010 to March 2011, with 20 (phase I) and 16 (phase II) participants. Additional file 1*:* Table S4 summarises the characteristics of the qualitative study participants (N = 36). Most participants were female, white, with between 1 and 10 DUs across both hands. In phase I, all activities and limitations evaluated in the CHFS were spontaneously brought up by participants as being difficult due to DUs, with the exception of ‘holding a bowl’ (Additional file 1: Table S5), while a number of additional concepts emerged in the discussion (Additional file 1: Table S6). Details of the modifications to the HDISS-DU are presented in Additional file 1: Table S7. The final 11 interviews of phase II suggested that the HDISS-DU instrument captures relevant issues and functional limitations relating to SSc-DUs in this patient population. Patients understood the instructions, items and response scales and found a 7-day recall period appropriate.

### Psychometric validation study

Participants (*N* = 517) were predominantly female (83.4%) and white (82.2%), most commonly with ≤ 3 DUs in total affecting both hands (Additional file 1*:* Table S8).

#### Final content validation

Detailed findings are described in Additional file 1: Result S9 and resulted in the removal of 2 items and amendment of the scoring system (from 0–5 to 1–6) and response scale, which was modified to include 2 additional response options, based on the data from the qualitative patient interviews and results of the psychometric analyses (Fig. 1 and Additional file 1: Table S7). The final HDISS-DU instrument was confirmed as 24 items in a single domain, with a 7-day recall period and 8 response options. Responses were scored from 1 to 6 (6 scores), where 1 is ‘yes, without difficulty’, 2 is ‘yes, with a little difficulty’, 3 is ‘yes, with some difficulty’, 4 is ‘yes with much difficulty’, 5 is ‘nearly impossible to do’ and ‘used unaffected hand only’ (both responses were assigned the same score), and 6 is ‘impossible’. The eighth response option ‘did not do this activity in the past 7 days’ was scored as missing. The overall HDISS-DU score was calculated as the mean of non-missing item scores (with a missing data threshold of < 12 items) and ranged from a minimum score of 1 to a maximum score of 6. Overall, the results support the content coverage and validity of the instrument.

#### Evaluation and measurement of psychometric properties.

##### Reliability

Ceiling (0%) and floor (range 1–3% depending on the week) effects were under minimal according to well-accepted criteria [23] (Additional file 1: Table S10). The HDISS-DU demonstrated excellent internal consistency reliability (Cronbach’s alpha 0.97–0.98), which was not improved by the removal of any individual item (Additional file 1: Table S11), as well as excellent test-retest reliability (ICCs > 0.80 in stable patients; Table 1).

**Table 1:** HDISS-DU score: test-retest reliability in stable patients

Definition of stable patients	Number	Mean HDISS-DU score (SD)			*T* value	*p* value	ICC [95% CI]
		Baseline	Week 4	Difference (week 4 − baseline)			
Patient-reported global change: ‘no change’ at week 4	170	2.83 (1.14)	2.84 (1.14)	0.01 (0.55)	0.33	0.7381	0.88 [0.85–0.91]
Physician-reported global change: ‘no change’ at week 4	151	2.79 (1.10)	2.82 (1.09)	0.03 (0.58)	0.60	0.5491	0.86 [0.81–0.89]
Patient-reported severity: identical score at baseline and week 4	178	2.80 (1.07)	2.84 (1.09)	0.04 (0.52)	1.02	0.3084	0.89 [0.85–0.91]
Physician-reported severity: identical score at baseline and week 4	273	2.92 (1.10)	2.91 (1.09)	− 0.01 (0.65)	− 0.18	0.8579	0.82 [0.78–0.86]

The reproducibility of HDISS-DU scores between baseline and week 4 was assessed in stable patients (defined in four different ways based on patient-reported and physician-reported global assessments) by ICCs and paired *t* tests

*CI* confidence interval, *DU* digital ulcer, *HDISS-DU* Hand Disability in Systemic Sclerosis-Digital Ulcers, *ICC* intraclass correlation coefficient, *n* number of participants, *SD* standard deviation

##### Construct validity

Ten convergent correlations were tested, and all were statistically significant (Table 2). Spearman’s rho ranged from 0.01 to 0.77 (median 0.63). Eight of ten convergent correlations were strong, and one was moderate, based on the standard criteria [19]. There was only a weak correlation between the number of DUs and HDISS-DU score (*r* 0.01–0.19). Eight discriminant correlations were tested, and again, all were statistically significant (Table 2). Half of these correlations were weak to moderate (depending on the measure and time point). However, there were moderate to strong correlations of the HDISS-DU score with presenteeism and overall work impairment for the WPAI-DU and with the SHAQ HAQ-DI arising domain score. There was also a strong correlation with the SHAQ HAQ-DI reach domain score (*r* 0.56–0.67). Each of the three tests for known-group validity was significant at week 16: HDISS-DU scores were significantly different in patients grouped by the number of DUs, DU complications category or the number of hands affected (Table 3). Tests for known-group validity were also significant for patients grouped by DU complications category at baseline and week 8.

**Table 2:** HDISS-DU score: construct validity

Measure	Validity assessed	Spearman’s rho		
		Baseline	Week 8	Week 16
Number of DUs	Convergent	0.01	0.10*	0.19**
SHAQ HAQ-DI grip domain score	Convergent	0.66***	0.71***	0.72***
SHAQ HAQ-DI hygiene domain score	Convergent	0.60***	0.64***	0.63***
SHAQ HAQ-DI eating domain score	Convergent	0.73***	0.77***	0.76***
SHAQ-DI dressing and grooming domain score	Convergent	0.69***	0.73***	0.73***
SHAQ VAS DU severity item score	Convergent	0.60***	0.60***	0.61***
Patient-reported severity	Convergent	0.57***	0.62***	0.64***
Physician-reported severity	Convergent	0.39***	0.37***	0.43***
WPAI:DU activity impairment	Convergent	0.63***	0.65***	0.72***
Global pain scale item score	Convergent	0.55***	0.61***	0.65***
SHAQ HAQ-DI arising domain score	Discriminant	0.39***	0.50***	0.50***
SHAQ HAQ-DI walking domain score	Discriminant	0.34***	0.47***	0.40***
SHAQ HAQ-DI reach domain score	Discriminant	0.56***	0.67***	0.63***
SHAQ VAS intestinal problems item score	Discriminant	0.23***	0.35***	0.33***
SHAQ VAS breathing problems item score	Discriminant	0.30***	0.39***	0.42***
WPAI:DU absenteeism	Discriminant	0.24*	0.26**	0.31**
WPAI:DU presenteeism	Discriminant	0.45***	0.55***	0.69**
WPAI:DU overall work impairment	Discriminant	0.45***	0.52***	0.66***

Spearman’s rank-order correlation assessed the association between the HDISS-DU score and other measures at baseline, week 8 and week 16. A priori hypotheses identified measures expected to capture similar concepts (assessed for convergent validity) and different concepts (assessed for discriminant validity) to the HDISS-DU score

*p < 0.05; **p < 0.01; ***p < 0.001

*DU* digital ulcer, *HAQ-DI* Health Assessment Questionnaire Disability Index, *HDISS-DU* Hand Disability in Systemic Sclerosis-Digital Ulcers, *SHAQ* Scleroderma Health Assessment Questionnaire, *VAS* visual analogue scale, *WPAI:DU* Work Productivity and Activity Impairment Questionnaire: Digital Ulcer

**Table 3:** HDISS-DU score: known-group validity

	Group	Number	Mean HDISS-DU score (SE)	*f* value/*t* value	*p* value
Baseline	Number of DUs				
	≤ 3	337	2.94 (0.06)	0.00	0.9854
	> 3	178	2.94 (0.08)		
	DU complications category				
	None	512	2.94 (0.05)	4.96	0.0263
	Mild	3	4.34 (0.63)		
	Moderate	0	N/A		
	Severe	0	N/A		
	Number of hands affected				
	One	184	3.02 (0.08)	1.32	0.2505
	Two	331	2.90 (0.06)		
Week 8	Number of DUs				
	≤ 3	276	2.75 (0.06)	2.39	0.1227
	> 3	152	2.92 (0.09)		
	DU complications category				
	None	407	2.76 (0.05)	8.96	0.0002
	Mild	20	3.75 (0.24)		
	Moderate	1	4.04 (1.05)		
	Severe	0	N/A		
	Number of hands affected				
	One	156	2.83 (0.09)	0.14	0.7133
	Two	227	2.87 (0.07)		
Week 16	Number of DUs				
	≤ 3	228	2.76 (0.07)	6.71	0.0100
	> 3	106	3.09 (0.10)		
	DU complications category				
	None	306	2.83 (0.06)	7.85	0.0005
	Mild	16	2.64 (0.27)		
	Moderate	0	N/A		
	Severe	12	4.05 (0.31)		
	Number of hands affected				
	One	134	2.74 (0.09)	6.10	0.0141
	Two	159	3.05 (0.08)		

Known-group validity was assessed by independent samples *t* test (2 patient groups; *t* value) or by ANOVA (≥ 3 patient groups; *f* value)

*ANOVA* analysis of variance, *DU* digital ulcer, *HDISS-DU* Hand Disability in Systemic Sclerosis-Digital Ulcers, *n* number of participants, *N/A* not applicable, *SE* standard error

##### Responsiveness

The analyses confirmed that HDISS-DU scores were significantly responsive to change in each of the five other measures tested (Table 4). Participants with increasing levels of improvement (indicated by the global assessments and responder status on the global pain scale [24]) also had improvements in HDISS-DU scores (indicated by reductions in the score), and vice versa. Effect sizes were small (0.0) for the no-change group and small/minimal change on the global assessment scales equated to small effect sizes, whereas larger changes corresponded with moderate to large effect sizes. The effect size was moderate for responders on the global pain scale. Conversely, the HDISS-DU was not sensitive to the cumulative number of new DUs (Additional file 1: Table S12).

**Table 4:** HDISS-DU score responsiveness

Response category	Response type	Number	LS mean change in HDISS-DU score^†^ (SE)	Overall *F* test value	*p* value	Effect size
Patient-reported severity score				18.04	< 0.0001	
− 6	Improvement	1	− 1.86 (0.72)			N/A
− 5	Improvement	5	− 1.41 (0.32)			− 1.3
− 4	Improvement	14	− 0.94 (0.19)			− 0.9
− 3	Improvement	52	− 0.66 (0.10)			− 0.6
− 2	Improvement	73	− 0.59 (0.08)			− 0.5
− 1	Improvement	114	− 0.22 (0.07)			− 0.2
0	No change	107	− 0.03 (0.07)			0.0
1	Deterioration	41	0.33 (0.11)			0.3
2	Deterioration	7	0.36 (0.27)			0.5
3	Deterioration	3	0.87 (0.42)			0.7
4	Deterioration	2	0.59 (0.51)			5.1
5	Deterioration	N/A	N/A			N/A
Physician-reported severity score				13.99	< 0.0001	
− 6	Improvement	2	− 0.66 (0.53)			− 2.2
− 5	Improvement	1	0.18 (0.75)			N/A
− 4	Improvement	9	− 0.99 (0.25)			− 1.1
− 3	Improvement	46	− 0.56 (0.11)			− 0.5
− 2	Improvement	90	− 0.47 (0.08)			− 0.4
− 1	Improvement	124	− 0.30 (0.07)			− 0.3
0	No change	129	− 0.02 (0.07)			− 0.0
1	Deterioration	25	0.11 (0.15)			0.2
2	Deterioration	7	1.09 (0.28)			0.8
3	Deterioration	2	0.63 (0.53)			0.6
4	Deterioration	N/A	N/A			N/A
5	Deterioration	1	0.29 (0.75)			N/A
Patient-reported global change score				28.25	< 0.0001	
1	Deterioration	7	0.58 (0.27)			0.4
2	Deterioration	18	0.72 (0.17)			0.6
3	Deterioration	33	0.18 (0.13)			0.2
4	No change	78	0.02 (0.08)			0.0
5	Improvement	111	− 0.25 (0.07)			− 0.3
6	Improvement	132	− 0.53 (0.06)			− 0.5
7	Improvement	40	− 0.86 (0.11)			− 0.8
Physician-reported global change score				23.56	< 0.0001	
1	Deterioration	3	1.18 (0.43)			6.3
2	Deterioration	21	0.55 (0.16)			0.4
3	Deterioration	40	0.10 (0.12)			0.2
4	No change	65	− 0.08 (0.09)			− 0.0
5	Improvement	122	− 0.17 (0.07)			− 0.2
6	Improvement	13	− 0.49 (0.07)			− 0.4
7	Improvement	55	− 0.63 (0.10)			− 0.7
Responder status				79.22	< 0.0001	
Responder^‡^	Improvement	169	− 0.65 (0.06)			− 0.6
Non-responder^§^	No change	251	0.01 (0.05)			0.0

Change from baseline to week 16 in HDISS-DU score was assessed by ANCOVA, controlling for baseline HDISS-DU score, by week 16 response for the global assessments and responder status (based on the 16-week change on the global pain scale). The effect size was calculated by subtracting the baseline scores from week 16 scores and dividing by the baseline score standard deviation

*ANCOVA* analysis of covariance, *HDISS-DU* Hand Disability in Systemic Sclerosis-Digital Ulcers, *n* number of participants, *SE* standard error

^†^A negative mean change in HDISS-DU score indicates an improvement

^‡^Defined as ≥ 3 point decrease in the score [24]

^§^Defined as < 3 point decrease in the score [24]

##### Minimal meaningful change threshold

Based on the comparisons with the global assessment scales, small improvements in DUs were associated with a 0.25–0.50 improvement in HDISS-DU score (Table 5), while a small deterioration on the global assessment scales was associated with a 0.18–0.19 reduction in HDISS-DU score.

**Table 5:** HDISS-DU score: anchor-based approach to identify a minimal meaningful change threshold

Measure	Anchor	Anchor response type	Number	Mean change in HDISS-DU score from baseline (SD)
Patient-reported severity	Improvement of less than one category	Less than minimal improvement	160	0.15 (0.70)
	Improvement of one category	Minimal improvement	114	− 0.25 (0.71)
	Improvement of more than one category	Greater than minimal improvement	145	− 0.71 (0.94)
Physician-reported severity	Improvement of less than one category	Less than minimal improvement	164	0.07 (0.75)
	Improvement of one category	Minimal improvement	124	− 0.29 (0.85)
	Improvement of more than one category	Greater than minimal improvement	148	− 0.55 (0.89)
Patient-reported global change	‘Minimally worse’	Minimal deterioration	33	0.19 (0.84)
	‘Minimally improved’	Minimal improvement	132	− 0.50 (0.85)
Physician-reported global change	‘Minimally worse’	Minimal deterioration	40	0.18 (0.76)
	‘Minimally improved’	Minimal improvement	130	− 0.50 (0.86)

Anchors and change in HDISS-DU score use week 16 data. A negative mean change in HDISS-DU score indicates an improvement

*HDISS-DU* Hand Disability in Systemic Sclerosis-Digital Ulcers, *n* number of participants, *SD* standard deviation

## Discussion

This research describes the comprehensive development, in accordance with FDA PRO guidance, and psychometric validation of the HDISS-DU, a new PROM that assesses the impact of DUs on hand function in patients with SSc-DUs. HDISS-DU development was based on the extensive patient input and appraised through qualitative interviews that support the content validation, concept saturation, face validity and ease of administration. Psychometric analyses support the excellent internal consistency reliability and test-retest reliability, satisfactory construct validity and high responsiveness of the HDISS-DU instrument in this patient population. The validated HDISS-DU instrument is available at https://eprovide.mapi-trust.org/instruments/hand-disability-in-systemic-sclerosis-digital-ulcers.

With the exception of the clinical assessments of DU number (weak) and physician-reported severity (moderate), convergent correlations between the HDISS-DU score and other measures expected to capture similar concepts were strong. The correlation between DU number and the HDISS score may have been weakened by the presence of DUs in other hand positions that were not recorded in DUAL-1 or DUAL-2 (only those from the proximal inter-phalangeal joint distally were recorded) [17] but might have influenced hand function. On the other hand, the weak correlation between DU number and the HDISS-DU score might indicate that, from patients’ perspectives, functional limitations in daily life activities are not directly associated with this simplified measure and that the position and/or severity of individual DUs has a greater influence on hand disability. Discriminant correlations were mainly of moderate strength, highlighting that the functional limitations relevant to patients and captured by the HDISS-DU are only partially captured by these other measures.

Here, we report an excellent internal consistency reliability for the HDISS-DU, with a Cronbach’s alpha of 0.97–0.98. In fact, this exceeds the recommended value of 0.90 [25], a score above which may indicate redundancy among items; however, Cronbach’s alpha is also influenced by the number of items and dimensionality [26]. For instance, the value of Cronbach’s alpha increases with more items regardless of the internal consistency [26]. We conducted a thorough, methodical, iterative evaluation of dimensionality and potential item redundancy that culminated in a decision to remove one redundant item based on the results of the psychometric analyses, together with data from the qualitative patient interviews and input from clinicians. Hence, the authors are confident that all items in the final HDISS-DU instrument are relevant to patients and not redundant. The higher than recommended value of Cronbach’s alpha may instead be due to the HDISS-DU being unidimensional and relatively long (24 items). It is important to note that Cronbach’s alpha is a property of the scores from a specific sample [26] and the value reported here applies to the DUAL-1 and DUAL-2 trial populations. The authors speculate that lower values of Cronbach’s alpha might be reported for more heterogeneous patient populations. Future studies will provide further insight into the performance of the HDISS-DU instrument.

To ensure suitability for use as a primary or secondary endpoint in clinical trials, development of the HDISS-DU has followed FDA guidance on PROs integrating patient input from the outset, using patients’ language, relevant recall timeframes and a simple response scale [10]. Our findings indicate that a preliminary minimal meaningful change in HDISS-DU score would be a 0.25–0.50 change, with an improvement of 0.50 constituting a conservative responder definition for future studies. Future research is necessary to recommend more robust thresholds for meaningful change.

The HDISS-DU is a disease-specific PROM developed and validated to assess hand function in patients with SSc-DUs. The integration of PROMs in clinical practice has the potential to improve patient care by screening for and identifying problems, monitoring progression and treatment response over time and identifying groups of patients with severe symptoms or limitations and those experiencing rapid deterioration [9]. Disease-specific PROMs are useful in complex conditions, such as SSc-DUs, where the content validity of broader generic PROMS (e.g. the EuroQol EQ-5D that examines aspects that fit a variety of different conditions) [27] may be questionable. While generic tools facilitate the comparison of disease and symptom burden across different disease populations, they vary in the extent to which items capture the experiences of specific patient populations and might miss items that patients with a specific disease consider important [28].

The HDISS-DU could be used in parallel with new clinical outcome measures currently in development, such as the DU clinical assessment score (DUCAS) for SSc [29]. The DUCAS is an objective, physician-defined measure that provides a composite clinical score for SSc-DUs; it was not developed with any patient input [29]. The use of the DUCAS or other similar clinical measures in conjunction with the HDISS-DU in a clinical trial setting should capture an objective clinical measure of disease severity and a patient-reported assessment of the impact of SSc-DUs on hand function, thus ensuring that patients’ perspectives are included in the evaluation of treatment response. It should be noted that, while the HDISS-DU captures patient-reported impacts of SSc-DUs on functional limitation to daily activities, it does not assess patients’ perceptions of their physical, mental or social well-being.

Some limitations of this research should be noted. Firstly, on a few occasions in qualitative interviews, patients mentioned that other aspects of their hand condition (e.g. scleroderma, curling of fingers, arthritis or lack of dexterity) were also related to difficulties in completing the activities discussed. However, the other aspects described tended to be constant, and thus, it was concluded that the measure is still likely to reflect functional changes associated with DUs. Another limitation is that factors related to the DUAL-1 and DUAL-2 trial designs or the disease natural history may have influenced the psychometric analyses. For instance, patients in both trials could take concomitant medications, e.g. pain relief [17], which may have weakened the correlations between scores on the global pain scale and the HDISS-DU. One final limitation that should be noted is the relatively complex relationship between DU activity and functional limitation, largely due to the DU location and number on the dominant or submissive hand, or both. However, SSc-DUs commonly affect both hands negating this issue, and during the HDISS-DU development, both the response scale and option ordering were optimised resulting in the amalgamation of response options ‘used unaffected hand only’ and ‘nearly impossible to do’.

## Conclusion

In conclusion, these psychometric analyses support the reliability, validity, discriminating ability and responsiveness to change of the HDISS-DU to capture the impact of DUs on hand function in this patient population in future clinical studies and clinical practice.

## Additional file

**Additional file 1: Method S1.** Major exclusion criteria in DUAL-1 and DUAL-2 [1]. **Method S2.** Additional methodological details for the final content validation. **Table S3.** Summary of the measures included for the final content validation of the HDISS-DU. **Table S4.** Baseline characteristics of patients in the qualitative research study (*N*=36). **Table S5.** Results of CHFS item assessment of the Phase I qualitative research study (*n*=20). **Table S6.** Additional concepts that emerged from discussions in the Phase I qualitative research study (*n*=20). **Table S7.** Summary of modifications made during the HDISS-DU development based on qualitative patient interviews (*N*=36). **Table S8.** Baseline characteristics of participants in the psychometric validation study (*N*=517). **Result S9.** Results of the final content validation. **Table S10.** Descriptive statistics for the HDISS-DU score. **Table S11.** HDISS-DU score: internal consistency reliability. **Table S12.** Responsiveness of the HDISS-DU score to the number of new DUs. **Table S13.** Confirmatory factor analysis for the HDISS-DU.

## Data Availability

Data are available upon reasonable request.
