# Supplementary material for: Psychometric validation of the Hand Disability in Systemic Sclerosis-Digital Ulcers (HDISS-DU®) patient-reported outcome instrument
Source: Arthritis Res Ther. 2020 Jan 6;22:3. doi: 10.1186/s13075-019-2087-4 (PMC6945532; doi:10.1186/s13075-019-2087-4)
Supplement: Supplementary file 1 — Additional file 1: Method S1. Major exclusion criteria in DUAL-1 and DUAL-2 [1]. Method S2. Additional methodological details for the final content validation. Table S3. Summary of the measures included for the final content validation of the HDISS-DU. Table S4. Baseline characteristics of patients in the qualitative research study (N=36). Table S5. Results of CHFS item assessment of the Phase I qualitative research study (n=20). Table S6. Additional concepts that emerged from discussions in the Phase I qualitative research study (n=20). Table S7. Summary of modifications made during the HDISS-DU development based on qualitative patient interviews (N=36). Table S8. Baseline characteristics of participants in the psychometric validation study (N=517). Result S9. Results of the final content validation. Table S10. Descriptive statistics for the HDISS-DU score. Table S11. HDISS-DU score: internal consistency reliability. Table S12. Responsiveness of the HDISS-DU score to the number of new DUs. Table S13. Confirmatory factor analysis for the HDISS-DU. [file 13075_2019_2087_MOESM1_ESM.docx]

**Psychometric validation of the Hand Disability in Systemic Sclerosis-Digital Ulcers (HDISS-DU®) patient-reported outcome instrument**

#

# **Supplementary material – Additional file 1**

## **Method S1**. Major exclusion criteria in DUAL-1 and DUAL-2.[1]

Major exclusion criteria included (i) digital ulcers due to other conditions than systemic sclerosis; (ii) the presence of other connective tissue diseases and (iii) the presence of any other pathology that could affect hand function or hand pain including patients affected to a severe degree (based on investigator discretion) by related conditions such as erosive arthritis, extensive calcinosis, and flexion contractures, as well as multiple amputations involving more than one digit. Patients provided written informed consent prior to their participation in the research study and relevant Institutional Review Boards approved all study protocols (including iterative amendments).

## Method S2. Additional methodological details for the final content validation

### Differential item functioning

Baseline descriptive statistics were compared for countries with >10 participants to explore potential outliers (differences in item means >1) that might indicate cultural differences. Differential item functioning was also explored in patients grouped by number of hands affected.

### Item redundancy

Highly correlated item pairs (r>0.80 in inter-item correlations; indicating that they may measure the same concept) were flagged for further investigation. Test-retest analyses (intraclass correlations, and paired t-test comparisons) were undertaken in ‘stable patients’. Stable patients were defined in two ways for these analyses: (1) patients who reported ‘no change’ at Week 4 on the patient-reported global assessment: global change in illness severity since baseline (patient-reported global change); and (2) patients who had the same rating at baseline and Week 4 for the patient-reported global assessment: severity of digital ulcers (DUs) (patient-reported severity).

Items under consideration for potential removal were tested for responsiveness using an analysis of covariance to compare change in HDISS-DU item scores, controlling for baseline item scores, by Week 16 scores in other measures and by responder status. Other measures included patient-reported severity, patient-reported global change, physician-reported assessment: severity of DU disease state (physician-reported severity), and physician-reported assessment: global improvement in DU disease state (physician-reported global change). Patients were grouped by responder status: responders (≥3 point decrease in hand pain) and non-responders (<3 point decrease in hand pain),[2] based on change at Week 16 from baseline on the global pain scale. Effect size was calculated for HDISS-DU item scores in patients grouped by responder status following US Food and Drug Administration guidance [3] by subtracting baseline scores from Week 16 scores, and dividing by the baseline score standard deviation.

### Response option ordering and redundancy

Items with fit residuals <–3 or >3 were flagged for potential removal and investigated further for test-retest reliability, responsiveness to change and effect size. Results from the Exploratory Factor Analysis and Rasch modelling informed the preliminary scoring algorithm. The potential for response option redundancy was explored by analysing item response option frequency distributions.

## Table S3. Summary of the measures included for the final content validation of the HDISS-DU

|  | **Number of items/description of endpoints** | **Response scale** | **Recall period** | **Timing of data collection in DUAL-1 and DUAL-2 [1]** | **Use in the present psychometric validation study** | | |
| --- | --- | --- | --- | --- | --- | --- | --- |
|  |  |  |  |  | **Outcome** | **Outcome range** | **Psychometric analysis** |
| **HDISS-DU [4]** | 24^†^ | Eight response options with six possible scores: 1 (‘yes without difficulty’) – 6 (‘impossible’)^†^ | 7 days | Baseline and Weeks 4, 8, 12 and 16 | HDISS-DU score: Arithmetic mean of non-missing item scores^‡^ | 1–6^†^ | All |
| **SHAQ [5]** | 20 in 8 functional domains from the HAQ-DI | 0 (‘without any difficulty’) – 3 (‘unable to do’) | 7 days | Baseline, Week 8 and Week 16 | SHAQ HAQ-DI domain score: Highest non-missing item score within the corresponding domain; SHAQ HAQ-DI total score: Arithmetic mean of non-missing domain scores^‡^ | 0–3 | Discriminant and convergent validity (individual domain scores) |
|  | 6 | Visual analogue scale: 0 (‘no symptoms’) – 100 (‘very severe symptoms’) on a 15 cm length scale; scores are then converted to a 0–3 scale using linear interpolation | 7 days | Baseline, Week 8 and Week 16 | SHAQ VAS item score;  SHAQ VAS total score: Arithmetic mean of non-missing scores^‡^ | 0–3 | Convergent validity (DU severity item score), discriminant validity (intestinal and breathing problems item scores) |
| **Patient-reported global assessment [6]** | 2 | Severity of DUs: 1 (‘not at all’) – 7 (‘extreme’) | 7 days | Baseline and Weeks 4, 8, 12 and 16 | Item score | 1–7 | Test-retest reliability, responsiveness, score interpretation, convergent validity |
|  |  | Global change in illness severity since baseline: 1 (‘very much worse’) – 7 (‘very much improved’) | 4–12 weeks^§^ | Weeks 4, 8, 12 and 16 | Item score | 1–7 | Test-retest reliability, responsiveness, score interpretation |
| **Physician-reported global assessment [6]** | 2 | Severity of DU disease state: 1 (‘not at all’) – 7 (‘extreme’) | 7 days | Baseline and Weeks 4, 8, 12 and 16 | Item score | 1–7 | Test-retest reliability, responsiveness, score interpretation, convergent validity |
|  |  | Global improvement in DU disease state since baseline: 1 (‘very much worse’) – 7 (‘very much improved’) | 4–12 weeks^§^ | Weeks 4, 8, 12 and 16 | Item score | 1–7 | Test-retest reliability, responsiveness, score interpretation |
| **WPAI:DU [7]** | 9 items from 5 topics | Mixed response scales | 7 days | Baseline, Week 8 and Week 16 | Absenteeism: Time missed from work (item score); presenteeism: Impairment while working (calculated from two item scores); overall work impairment (calculated from multiple item scores); activity impairment (item score) | 0–100%, with higher numbers indicting greater impairment/lower productivity | Convergent validity (activity impairment), discriminant validity (absenteeism, presenteeism and overall work impairment) |
| **Global pain scale [6, 8]** | 1 (worst pain) | 1 (‘no pain’) – 10 (‘worst pain imaginable’) | 7 days | Baseline and Weeks 4, 8, 12 and 16 | Item score | 1–10 | Convergent validity, responsiveness |
| **Clinical assessment: DU complications** | Number and type of complication(s) | N/A | Current | All visits and between visits | DU complication category | None, mild,^¶^ moderate,^††^ severe^‡‡^ | Known-group validity |
| **Clinical assessment: Evaluation of DUs** | Number of existing or new of DU(s) | N/A | Current | All visits | Number of DUs; number of new DUs; number of hands affected | Continuous | Convergent validity (number of DUs), known-group validity (number of hands affected, number of DUs), responsiveness (cumulative number of new DUs) |

^†^Participants completed a 26-item questionnaire in DUAL-1 and DUAL-2 and this was refined to 24 items during final content validation in the present psychometric validation study, while the response scale used in these trials (0=‘without difficulty’, 1=‘little difficulty’; 2=‘some difficulty’; 3=‘much difficulty’; 4=‘nearly impossible’; 5=‘impossible’; 6=’did not do activity in the past 7 days’; and 7=‘used unaffected hand only’) was also refined to 1–6 during final content validation, with ‘used affected hand only’ scored as a 5 and ‘did not do activity in the past 7 days’ scored as missing.

^‡^For the HDISS-DU score, if ≥12 item scores are missing, the score is considered as missing; for the SHAQ HAQ-DI total score: If ≥3 domain scores are missing, the total score is considered as missing; for the SHAQ VAS total score: If ≥2 item scores are missing, the total score is considered missing.

^§^Determined by the number of weeks since the baseline visit for each time point.

^¶^Requiring (i) class I–III narcotics or >50% increase in dose (compared with baseline) OR (ii) initiation of systemic antibiotics.

^††^Requiring (i) AND (ii) (see above footnote^¶^).

^‡‡^Any of the following: critical ischaemic crisis necessitating hospitalisation; gangrene or auto amputation; surgical and chemical sympathectomy, vascular reconstructions, or any unplanned surgery in the management of hand SSc manifestation(s); use of parenteral prostanoids; or use of endothelin receptor antagonists. The presence of a severe complication resulted in permanent discontinuation of the study medication

DU, digital ulcer; HAQ-DI, Health Assessment Questionnaire Disability Index; N/A, not applicable; SHAQ, Scleroderma Health Assessment Questionnaire; VAS, visual analogue scale; WPAI:DU, Work Productivity and Activity Impairment Questionnaire: Digital Ulcer (version 2.0).

## Table S4. Baseline characteristics of patients in the qualitative research study (N=36)

|  | **Phase I**  **n=20** | **Phase II**  **n=16** |
| --- | --- | --- |
| Age, years, mean (SD) [range] | 47.3 (11.1) [18–64] | 59.1 (10.3) [39–73] |
| Female, n (%) | 17 (85.0) | 12 (75.0) |
| Hispanic or Latino ethnicity, n (%) | 1 (5.0) | 2 (12.5) |
| Race^†^, n (%) |  |  |
| White | 15 (75.0) | 15 (93.8) |
| American Indian or Alaska Native | 1 (5.0) | 0 (0.0) |
| Black or African American | 4 (20.0) | 0 (0.0) |
| Other | 2 (10.0) | 0 (0.0) |
| Missing | 0 (0.0) | 1 (6.3) |
| Employment, n (%) |  |  |
| Full-time | 8 (40.0) | 1 (6.3) |
| Part-time | 2 (10.0) | 2 (12.5) |
| Homemaker | 2 (10.0) | 1 (6.3) |
| Retired | 2 (10.0) | 7 (43.7) |
| Disabled | 5 (25.0) | 5 (31.2) |
| Other | 1 (5.0) | 0 (0.0) |
| Number of active DUs across both hands, n (%) | | |
| 0 | 0 (0.0) | 0 (0.0) |
| 1 | 3 (15.0) | 6 (37.5) |
| 2 | 12 (60.0) | 3 (18.8) |
| 3 | 2 (10.0) | 1 (6.3) |
| >3^‡^ | 2 (10.0) | 6 (37.5) |
| Missing | 1 (5.0) | 0 (0.0) |
| Number of active DUs on dominant hand, n (%) | | |
| 0 | 3 (15.0) | 1 (6.3) |
| 1 | 10 (50.0) | 10 (62.5) |
| 2 | 5 (25.0) | 1 (6.3) |
| 3 | 1 (5.0) | 2 (12.5) |
| >3^§^ | 0 (0.0) | 2 (12.5) |
| Missing | 1 (5.0) | 0 (0.0) |
| Number of active DUs on submissive hand, n (%) | | |
| 0 | 5 (25.0) | 5 (31.2) |
| 1 | 9 (45.0) | 5 (31.2) |
| 2 | 3 (15.0) | 3 (18.8) |
| 3 | 2 (10.0) | 2 (12.5) |
| >3^§^ | 0 (0.0) | 1 (6.3) |
| Missing | 1 (5.0) | 0 (0.0) |
| Type of DUs, n (%) |  |  |
| Vascular only | 17 (85.0) | 13 (81.3) |
| Vascular and other | 3 (15.0) | 2 (12.5) |
| Missing | 0 (0.0) | 1 (6.3) |
| SHAQ HAQ-DI total score [scale 0–3], mean (SD) [range] | 0.90 (0.60) [0.00­­–2.25] | 1.10 (0.73) [0.13–2.38] |
| Patient-reported severity [scale 1–7], mean (range) | 4.00 (1–7) | 4.00 (2–7) |
| Global pain scale [scale 1–10], mean (range) | 5.20 (1–10) | 5.25 (2–10) |

^†^Not mutually exclusive.

^‡^Phase I range: 5–6; Phase II range 4–10.

^§^Phase II participants with >3 DUs had 5 DUs on the respective hand.

%, percentage of participants; DU, digital ulcer; HAQ-DI, Health Assessment Questionnaire Disability Index; n, number of participants; SD, standard deviation; SHAQ, Scleroderma Health Assessment Questionnaire.

## Table S5. Results of CHFS item assessment of the Phase I qualitative research study (n=20)

| **Item** | **CHFS score,^†^ mean (SD)** | **Clearly understood, n/N (%)** | **Difficulty due to DUs, n/N (%)** | **Concept emerged in initial discussions, n (%)** |
| --- | --- | --- | --- | --- |
| 1. Hold bowl | 0.95 (0.89) | 20/20 (100) | 20/20 (100) | 0 (0) |
| 1. Seize full bottle and raise it | 1.15 (1.14) | 18/20 (90) | 19/20 (95) | 2 (10) |
| 1. Hold plate full of food | 1.05 (1.19) | 20/20 (100) | 16/20 (80) | 3 (15) |
| 1. Pour liquid from bottle into jar | 1.15 (1.09) | 18/19 (95) | 18/19 (95) | 3 (15) |
| 1. Unscrew lid from jaw | 1.80 (1.32) | 18/19 (95) | 18/19 (95) | 11 (55) |
| 1. Cut meat with knife | 1.60 (1.39) | 19/19 (100) | 19/19 (100) | 15 (75) |
| 1. Prick things well with fork | 0.95 (1.23) | 17/20 (85) | 17/20 (85) | 2 (10) |
| 1. Peel fruit | 2.00 (1.52) | 20/20 (100) | 20/20 (100) | 1 (5) |
| 1. Button shirt | 2.15 (1.46) | 19/19 (100) | 18/19 (95) | 11 (55) |
| 1. Open and close zipper | 1.90 (1.48) | 19/19 (100) | 18/19 (95) | 11 (55) |
| 1. Squeeze new tube of toothpaste | 1.10 (1.45) | 20/20 (100) | 19/20 (95) | 2 (10) |
| 1. Hold toothbrush efficiently | 1.30 (1.34) | 19/20 (95) | 18/20 (90) | 7 (35) |
| 1. Write short sentence | 1.15 (1.27) | 19/19 (100) | 14/19 (74) | 8 (40) |
| 1. Write letter | 1.60 (1.39) | 19/20 (95) | 20/20 (100) | 8 (40) |
| 1. Turn round door knob | 1.35 (1.31) | 20/20 (100) | 19/20 (95) | 3 (15) |
| 1. Cut piece of paper with scissors | 1.50 (1.40) | 20/20 (100) | 20/20 (100) | 6 (30) |
| 1. Pick up coins from table top | 2.40 (1.57) | 20/20 (100) | 9/20 (45) | 6 (30) |
| 1. Turn key in lock | 1.70 (1.56) | 20/20 (100) | 19/20 (95) | 2 (10) |

^†^Scoring of response options based on a 0–5 scale: 0= ‘yes, without difficulty’; 1= ‘yes, with a little difficulty’; 2= ‘yes, with some difficulty’; 3= ‘yes, with much difficulty’; 4= ‘nearly impossible to do’; 5= ‘impossible’. Higher scores indicate decreased hand functioning over the last week.

%, percentage of participants with an affirmative response of those who provided feedback; CHFS, Cochin Hand Function Scale; DU, digital ulcer; n, number of participants with an affirmative response; N, number of participants providing feedback; SD, standard deviation.

## Table S6. Additional concepts that emerged from discussions in the Phase I qualitative research study (n=20)

| **Concept** | **n** | **%** |
| --- | --- | --- |
| Using finger tips to manipulate (e.g. typing on a keyboard) | 15 | 75 |
| Washing dishes (e.g. getting hands wet is painful | 15 | 75 |
| Cleaning (e.g. vacuuming) | 14 | 70 |
| Other dressing (e.g. putting on jewellery) | 12 | 60 |
| Fitness activities or hobbies (e.g. reading) | 12 | 60 |
| Bathing/showering (e.g. washing hair) | 10 | 50 |
| Other grooming/toileting (e.g. using toilet paper) | 10 | 50 |
| Touching/washing linens or fabrics (e.g. folding laundry) | 9 | 45 |
| Eating food (e.g. using utensils) | 8 | 40 |
| Cooking food (e.g. washing food) | 8 | 40 |
| Sleeping | 6 | 30 |
| Driving (e.g. opening car door) | 4 | 20 |

%, percentage of participants; n, number of participants.

## **Table S7.** Summary of modifications made during the HDISS-DU development based on qualitative patient interviews (N=36)

|  | **Phase I** | | **Phase II** | | **Final instrument** |
| --- | --- | --- | --- | --- | --- |
|  | **Modifications** | **Rationale** | **Modifications** | **Rationale** |  |
| **Instructions** | | | | | |
| **Answer the following questions regarding your ability without the help of any assistive device during the last week:** | **Thinking about using your hand(s) affected by ulcers on your fingers, please answer the questions below.**  **If you were ONLY able to complete the activity by using an unaffected hand or by wearing gloves please select the answer choice “Not relevant / Did not do this activity in the past 7 days.”**  **Please consider your ability to do these activities over the past 7 days.**  *[instructions repeated on top of each page]* | *Specify hand(s) with ulcers:* Some participants noted difficulty in differentiating their limitations as related to DU(s) specifically versus scleroderma.  *Assistive devices removed:* Participants did not report common use of assistive devices due to DU(s) in general so this language removed.  *Response option information added:* Participants reported using an unaffected hand and/or gloves for some activities, so clarification made for respondents to respond by choosing the new response option.  *Recall period wording revised:* “Past 7 days” instead of “last week” based on translation expert review (e.g., last week doesn’t always mean the past 7 days, when translated, so recommend past 7 days to be clear). | **Thinking about using your hand(s) affected by ulcers on your fingers, please answer the questions below.**  **Please consider your ability to do these activities over the past 7 days.**  **If you were only able to complete the activity by using an unaffected hand, or if you did not do this activity, please check the box that says “Used unaffected hand only or did not do this activity.”**  *[instructions not repeated on top of each page as most participants noted that they did not reference the instructions after reading them on the first page]* | *Underlining of specific terms:* For emphasis during participant review of instructions.  *Re-ordering of sections:* To streamline the order of instruction comprehension.  *Gloves removed:* Participants reported choosing a response for some items while thinking of using gloves to perform the activity, however they did not choose the appropriate response option, as per the instructions. In addition to modification of instructions, term “bare hands” was included for certain items to clarify and remove potential impact of wearing gloves.  *Response option information revised:* Participants reported sometimes compensating by using an unaffected hand or not performing an activity within the appropriate timeframe, so clarification made for respondents to respond by choosing the new response option. | **Thinking about using your hand(s) affected by ulcers on your fingers, please answer the questions below.**  **Please consider your ability to do these activities over the past 7 days.**  **If you did not complete the activity in the past 7 days, please check the box that says “Did not do this activity in the past 7 days.”**  **If you were only able to complete the activity by using an unaffected hand, please check the box that says “Used unaffected hand only.”**  *(Rationale for final changes: Some participants were skipping over or not noticing the “Did not do activity” portion of the instructions. The order was changed to have did not do this activity first as unaffected hand applies to a smaller proportion of participants.*  *“Used unaffected hand / Did not do activity” separated into 2 answer choices to help with clarity and ensure that participants noted both choices.*  *Also, “in the past 7 days” was added back into the “Did not do this activity in the past 7 days” response option to remind patients of the recall period.*  *The wording of the portion of the instructions associated with these last two response options was split into two sentences for clarity.* |
| **Response Options** | | | | | |
| Yes, without difficulty (0);  Yes, with a little difficulty (1);  Yes, with some difficulty (2);  Yes, with much difficulty (3);  Nearly impossible to do (4);  Impossible (5) | **Addition of 7^th^ response option:**  Not relevant/ Did not do this activity in past 7 days (6) | Some participants had not completed activities listed on questionnaire in last week; further, some activities asked about in the questionnaire were so difficult, patients reported not doing these activities or reported using an unaffected hand without DU(s). | **Modification of 7^th^ response option:**  Used unaffected hand only or did not do this activity (6) | Participant comprehension of the previous response option wording, as related to the instructions, was not appropriate (i.e., participants sometimes responded to items while thinking about wearing gloves); new wording expected to be more easily comprehended. | Yes, without difficulty (0);  Yes, with a little difficulty (1);;  Yes, with some difficulty (2);  Yes, with much difficulty (3);  Nearly impossible to do (4);  Impossible (5); Did not do this activity in the past 7 days (6)  Used unaffected hand only (7) |
| **Heading Names** | | | | | |
| C1-In the Kitchen; items 1–8 C2-Dressing; items 9–10 C3-Hygiene; items 11–12 C4-In the Office; items 13–14 C5-Other; items 15–18 | Removal of heading names | Many participants reported that the headings were not necessary and not always mutually exclusive (e.g., activities “in the office” might occur in other settings and were not necessarily work related).  Also, with the addition of 9 new items for Phase 2, items did not always fit well within specific categories. | N/A | N/A | No headings |
| **Items** | | | | | |
| 1. Can you hold a bowl? | Delete item | Deletion because activity so similar to item 3. |  |  |  |
| 2. Can you seize a full bottle and raise it? | Delete item | Deletion because activity so similar to item 4. In addition, some difficulty was reported around the word “seize.” |  |  |  |
| 3. Can you hold a plate full of food? | 1. Can you hold a plate full of food using your fingers? | Participants reported ability to compensate when completing this activity by using palms of hands instead of using DU-affected fingers, thus language added to clarify use of fingers. | N/A | N/A | 1. Can you hold a plate full of food using your fingers? |
| 4. Can you pour liquid from a bottle into a glass? | 2. Can you pour liquid from a large full bottle into a glass or cup? | Participants noted the difference between different sizes, shapes, and fullness of bottles, so further details were added to clarify. Also, participants noted pouring into both cups as well as glasses, so specific language added to address. | N/A | N/A | 2. Can you pour liquid from a large full bottle into a glass or cup? |
| 5. Can you unscrew the lid from a jar opened before? | 3. Can you use your hands to unscrew the lid from a jar that has previously been opened? | Participants noted using jar openers to help with this activity, thus the term “use your hands” was added for clarification.  Some difficulty understanding the term “jar opened before,” so slight clarification in language was made to address this. | 3. Can you use your bare hands to unscrew the lid from a jar that has previously been opened? | Some participants reported sometimes using assistive devices (e.g., opener) to perform this activity, so “bare” added to clarify that responses should be chosen based on using bare hands only. | 3. Can you use your bare hands to unscrew the lid from a jar that has previously been opened? |
| 6. Can you cut meat with a knife? | 4. Can you cut food with a knife? | Translation expert review suggested that the term “meat” was too specific, thus the modification was made to the more general term “food.” | N/A | N/A | 4. Can you cut food with a knife? |
| 7. Can you prick things well with a fork? | 5. Can you use a fork to eat your food? | Participants noted some difficulty with the word “prick,” thus clarification made to address. | N/A | N/A | 5. Can you use a fork to eat your food? |
| 8. Can you peel fruit? | Delete item | Deletion because not a frequently undertaken activity (i.e., not all participants had peeled fruit in the past 7 days), and activity was seldom mentioned in emergent results; also participants reported variability in types of fruit and using assistive tools (e.g., knife, peeler) to help complete the activity. |  |  |  |
|  | 6. Can you prepare food with your hands? | Commonly reported activity, noted in emergent questions, as relevant to DU-related hand functioning. | 6. Can you prepare food with your bare hands? | Some participants reported sometimes using assistive devices (e.g., gloves, cooking utensils) to perform this activity, so “bare” added to clarify that responses should be chosen based on using bare hands only. | 6. Can you prepare food with your bare hands? |
|  | 7. Can you wash dishes in the sink with your hands? | Commonly reported activity, noted in emergent questions, as relevant to DU-related hand functioning. | 7. Can you wash dishes in the sink with your bare hands? | Some participants reported sometimes using assistive devices (e.g., gloves) to perform this activity, so “bare” added to clarify that responses should be chosen based on using bare hands only. | 7. Can you wash dishes in the sink with your bare hands? |
| 9. Can you button your shirt? | 8. Can you button your clothing? | Participants reported that “shirt” was too specific, and not all participants reported wearing shirt with buttons. | N/A | N/A | 8. Can you button your clothing? |
| 10. Can you open and close a zipper? | New item number 9; no change to item wording. |  | N/A | N/A | 9. Can you open and close a zipper? |
|  | 10. Can you pull on your socks? | Commonly reported activity, noted in emergent questions, as relevant to DU-related hand functioning. | N/A | N/A | 10. Can you pull on your socks? |
|  | 11. Can you tie your shoelaces? | Commonly reported activity, noted in emergent questions, as relevant to DU-related hand functioning. | N/A | N/A | 11. Can you tie your shoelaces? |
| 11. Can you squeeze a new tube of toothpaste? | 12. Can you squeeze a tube of toothpaste with your fingers? | Deletion of “new” when describing the state of the tube, given that participants reported new tube poses less difficulty.  Participants reported ability to compensate when completing this activity instead of using DU-affected fingers, thus language added to clarify use of fingers. | N/A | N/A | Delete item due to too much variability inherent in the activity. Participants reported variability related to different types of toothpaste tubes, varying difficulty depending on how full the tube is, difficulties with opening the tube which was not the intent of the question, and multiple compensatory strategies. |
| 12. Can you hold a toothbrush efficiently? | 13. Can you hold a toothbrush to brush your teeth? | It was noted that solely the act of holding the toothbrush poses less difficulty than holding the toothbrush to complete the activity of brushing one’s teeth.  Some difficulty understanding the term “efficiently,” thus clarification in language was made to address this. | N/A | N/A | 13. Can you hold a toothbrush to brush your teeth? |
|  | 14. Can you wash your hands with soap and water? | Commonly reported activity, noted in emergent questions, as relevant to DU-related hand functioning. | N/A | N/A | 14. Can you wash your hands with soap and water? |
|  | 15. Can you wash yourself in the shower or bath with your hands? | Commonly reported activity, noted in emergent questions, as relevant to DU-related hand functioning. | 15. Can you wash yourself in the shower or bath with your bare hands? | Some participants reported sometimes using assistive devices (e.g., gloves) to perform this activity, so “bare” added to clarify that responses should be chosen based on using bare hands only. | 15. Can you wash yourself in the shower or bath with your bare hands? |
|  | 16. Can you brush your hair? | Commonly reported activity, noted in emergent questions, as relevant to DU-related hand functioning. | N/A | N/A | 16. Can you brush your hair? |
|  | 17. Can you put cream or lotion on your face? | Commonly reported activity, noted in emergent questions, as relevant to DU-related hand functioning. | 17. Can you put cream or lotion on your face with your bare hands? | Some participants reported sometimes using assistive devices (e.g., gloves) to perform this activity, so “with your bare hands” added to clarify that responses should be chosen based on using bare hands only. | 17. Can you put cream or lotion on your face with your bare hands? |
| 13. Can you write a short sentence with a pencil or ordinary pen? | 18. Can you write a short sentence with a pencil or pen? | Deletion of term “ordinary” as not necessary. | N/A | N/A | 18. Can you write a short sentence with a pencil or pen? |
| 14. Can you write a letter with a pencil or ordinary pen? | Delete item | Deletion because so similar to item 13 and because less relevant (i.e., participants reported more commonly writing longer letters/ documents electronically). |  |  |  |
|  | 19. Can you use a keyboard to type? | Commonly reported activity, noted in emergent questions, as relevant to DU-related hand functioning. | N/A | N/A | 19. Can you use a keyboard to type? |
|  | 20. Can you use your finger tips to press buttons on household appliances? | Commonly reported activity, noted in emergent questions, as relevant to DU-related hand functioning. | N/A | N/A | 20. Can you use your finger tips to press buttons on household appliances? |
|  | 21. Can you use your finger tips to press small buttons, such as those on a cell phone? | Commonly reported activity, noted in emergent questions, as relevant to DU-related hand functioning. | N/A | N/A | 21. Can you use your finger tips to press small buttons, such as those on a cell phone? |
| 15. Can you turn a round door knob? | 22. Can you open a door? | Participants noted that “round door knob” was too specific, often using different shapes of door knobs other than round (such as lever-style handles), so modification of wording was made to address this and make it more general and relevant. Translation expert review confirmed this change. | N/A | N/A | 22. Can you open a door? |
| 16. Can you cut a piece of paper with scissors? | 24. Can you use scissors to cut something? | Participant report and translation expert review suggested that “piece of paper” was too specific, thus the modification was made to the more general term “something.” | N/A | N/A | 24. Can you use scissors to cut something? |
| 17. Can you pick up coins from a table top? | 25. Can you pick up coins using your finger tips? | Participants reported ability to compensate when completing this activity by sliding or sweeping coins off of a tabletop instead of using DU-affected fingers, thus language added to clarify use of fingers. | N/A | N/A | 25. Can you pick up coins using your finger tips? |
| 18. Can you turn a key in a lock? | New item 23; no change to item wording. | N/A | N/A | N/A | 23. Can you turn a key in a lock? |
|  | 26. Can you sweep the floor with a broom? | Commonly reported activity, noted in emergent questions, as relevant to DU-related hand functioning. | N/A | N/A | 26. Can you sweep the floor with a broom? |
|  | 27. Can you grip the steering wheel to drive a car? | Commonly reported activity, noted in emergent questions, as relevant to DU-related hand functioning. | N/A | N/A | 27. Can you grip the steering wheel to drive a car? |

DU, digital ulcers; HDISS-DU, Hand Disability in Systemic Sclerosis-Digital Ulcers; N/A, not applicable.

## Table S8. Baseline characteristics of participants in the psychometric validation study (N=517)

| **Characteristic** | **Screening/baseline** |
| --- | --- |
| Age, mean (SD) [range] | 50.5 (12.8) [18–81] |
| Female, n (%) | 431 (83.4) |
| Ethnicity/Race^†^, n (%) |  |
| Asian | 22 (4.3) |
| Black or African American | 6 (1.2) |
| Hispanic | 39 (7.5) |
| White or Caucasian | 425 (82.2) |
| Other | 19 (3.7) |
| Missing | 6 (1.2) |
| SSc serology, n (%) |  |
| Diffused | 287 (55.5) |
| Limited | 229 (44.3) |
| Number of DUs, n (%) |  |
| 0–3 | 337 (65.4) |
| ≥4 | 178 (34.6) |
| Number of hands affected, n (%) |  |
| 0 | 0 (0.0) |
| 1 | 184 (35.7) |
| 2 | 331 (64.3) |
| DU complications, n (%) |  |
| None | 512 (99.4) |
| Mild^‡^ | 3 (0.6) |
| Moderate^§^ | 0 (0.0) |
| Severe^¶^ | 0 (0.0) |

^†^Not mutually exclusive.

^‡^Requiring (i) class I–III narcotics or >50% increase in dose (compared with baseline) OR (ii) initiation of systemic antibiotics.

^§^Requiring (i) AND (ii) (see above footnote ^‡^).

^¶^Any of the following: critical ischaemic crisis necessitating hospitalisation; gangrene or auto amputation; surgical and chemical sympathectomy, vascular reconstructions, or any unplanned surgery in the management of hand SSc manifestation(s); use of parenteral prostanoids; or use of endothelin receptor antagonists. The presence of a severe complication resulted in permanent discontinuation of the study medication.

%, percentage of participants; DU, digital ulcer; n, number of participants; SD, standard deviation; SSc, systemic.

## Result S9. Results of the final content validation

These analyses suggest that there is no evidence of differential item functioning either by country or by the number of hands affected by digital ulcers (DUs). Results pointed towards a single domain as most appropriate for the HDISS-DU. Although the Exploratory Factor Analysis (EFA) initially highlighted a four-factor solution, the first factor had an eigenvalue >13, explained over 50% of the variance and demonstrated the highest item loading (with the exception of one item), therefore, a single factor (~domain) structure was taken forward. Items were moderately to highly correlated with one another (Spearman’s rho: 0.20–0.84, all p<0.0001). Four highly correlated item pairs were flagged for further investigated (Spearman’s rho>0.80). However, these demonstrated excellent test-retest reliability (all intraclass correlations ≥0.65), with moderate to large effect sizes (≥0.50 and ≥0.80, respectively) suggesting that these items are responsive and hence, a decision was made to keep three of the item pairs. The final item pair (‘open a door’ and ‘turn a key’) also had highly correlated residuals in Rasch modelling suggesting redundancy and a decision was made to remove ‘open a door’ from the HDISS-DU. A second item (‘grip a steering wheel’) was removed due to poor fit in the EFA and Rasch modelling, in addition to a high degree of missingness (a large proportion of participants responded that they ‘did not do this activity in the past 7 days’). This meant that the 26-item HDISS-DU was refined from 26 to 24 items (**Figure 1**).

In general, most response options were regularly used with low ceiling or floor effects suggesting that most items are relevant, everyday activities. However, the options, ‘used unaffected hand only’ and ‘did not do this activity in the past 7 days’ were infrequently used and Rasch modelling suggested response mis-ordering in their previous positions. A decision was made to refine the 8 response options to 6 by combining ‘nearly impossible to do’ and ‘used unaffected hand only’, and considering ‘did not do this activity in the past 7 days’ as missing.

The Confirmatory Factor Analysis confirmed good overall fit of the single factor (~domain) solution with all item factor loadings >0.75 and comparative fit indices ranging from 0.94–0.97 at baseline, Week 8 and Week 16 **(Supplementary Table S13)**. A mean score of non-missing items was confirmed as appropriate (stable over the 16-week study period, with no ceiling effects and only minor floor effects) and a missing data threshold of <12 items.

## Table S10. Descriptive statistics for the HDISS-DU score

|  | **Baseline**  **(n=517)** | **Week 4**  **(n=494)** | **Week 8**  **(n=460)** | **Week 12**  **(n=427)** | **Week 16**  **(n=441)** |
| --- | --- | --- | --- | --- | --- |
| Mean score (SD) | 2.9 (1.09) | 2.9 (1.09) | 2.8 (1.08) | 2.7 (1.08) | 2.7 (1.11) |
| Mean change in score from baseline (SD) | N/A | –0.1 (0.67) | ­–0.2 (0.78) | –0.3 (0.79) | –0.2 (0.87) |
| Median | 2.8 | 2.8 | 2.6 | 2.5 | 2.6 |
| Range | 1.0–5.9 | 1.0–5.8 | 1.0–5.6 | 1.0–5.6 | 1.0–5.8 |
| Floor, n (%) | 8 (1.5) | 7 (1.4) | 7 (1.5) | 12 (2.8) | 13 (2.9) |
| Ceiling, n (%) | 0 (0.0) | 0 (0.0) | 0 (0.0) | 0 (0.0) | 0 (0.0) |

A negative mean change in HDISS-DU score indicates an improvement.

%, percentage of participants; HDISS-DU, Hand Disability in Systemic Sclerosis-Digital Ulcers; n, number of participants; N/A, not applicable; SD, standard deviation.

## Table S11. HDISS-DU score: internal consistency reliability

| **HDISS-DU item score** | **Baseline (n=517)** | | **Week 8 (n=460)** | | **Week 16 (n=441)** | |
| --- | --- | --- | --- | --- | --- | --- |
|  | **Cronbach’s alpha** | **Cronbach’s alpha^†^** | **Cronbach’s alpha** | **Cronbach’s alpha^†^** | **Cronbach’s alpha** | **Cronbach’s alpha^†^** |
| HDISS-DU score | 0.969 |  | 0.976 |  | 0.978 |  |
| Hold a plate |  | 0.968 |  | 0.975 |  | 0.977 |
| Pour liquid |  | 0.968 |  | 0.975 |  | 0.977 |
| Unscrew lid |  | 0.968 |  | 0.975 |  | 0.977 |
| Cut food |  | 0.968 |  | 0.974 |  | 0.977 |
| Use fork |  | 0.968 |  | 0.975 |  | 0.977 |
| Prepare food |  | 0.968 |  | 0.975 |  | 0.977 |
| Wash dishes |  | 0.969 |  | 0.975 |  | 0.978 |
| Button clothing |  | 0.968 |  | 0.974 |  | 0.977 |
| Open/close zipper |  | 0.968 |  | 0.974 |  | 0.977 |
| Pull on socks |  | 0.968 |  | 0.975 |  | 0.977 |
| Tie shoelaces |  | 0.968 |  | 0.975 |  | 0.977 |
| Hold toothbrush |  | 0.968 |  | 0.975 |  | 0.977 |
| Wash hands |  | 0.968 |  | 0.975 |  | 0.977 |
| Shower or bath |  | 0.968 |  | 0.975 |  | 0.977 |
| Brush hair |  | 0.968 |  | 0.975 |  | 0.977 |
| Put cream on face |  | 0.968 |  | 0.975 |  | 0.977 |
| Write short sentence |  | 0.968 |  | 0.975 |  | 0.977 |
| Type on keyboard |  | 0.969 |  | 0.975 |  | 0.977 |
| Press buttons |  | 0.968 |  | 0.975 |  | 0.977 |
| Press cell phone |  | 0.969 |  | 0.975 |  | 0.977 |
| Turn a key |  | 0.968 |  | 0.974 |  | 0.977 |
| Use scissors |  | 0.968 |  | 0.974 |  | 0.977 |
| Pick up coins |  | 0.968 |  | 0.975 |  | 0.977 |
| Sweep floor |  | 0.968 |  | 0.975 |  | 0.977 |

HDISS-DU score reliability was assessed by Cronbach’s alpha at baseline, Week 8 and Week 16. Analyses were repeated with each items removed separately to establish if reliability would be improved through removal of an item.

^†^If item deleted.

HDISS-DU, Hand Disability in Systemic Sclerosis-Digital Ulcers; n, number of participants.

## Table S12. Responsiveness of the HDISS-DU score to the number of new DUs

|  | **Cumulative number of new DUs at Week 16** | | | | | | | |
| --- | --- | --- | --- | --- | --- | --- | --- | --- |
|  | **0**  **(n=247)** | **1**  **(n=71)** | **2**  **(n=43)** | **3**  **(n=26)** | **4**  **(n=18)** | **5**  **(n=17)** | **6**  **(n=7)** | **7**  **(n=12)** |
| Mean change in HDISS-DU score from baseline (at Week 16) | –0.34 | –0.23 | –0.01 | 0.03 | –0.30 | 0.00 | 0.12 | 0.17 |

A negative mean change in HDISS-DU score indicates an improvement.

DU, digital ulcer; HDISS-DU, Hand Disability in Systemic Sclerosis-Digital Ulcers; n, number of participants.

## Table S13. Confirmatory factor analysis for the HDISS-DU

| **HDISS-DU item score** | **Factor 1 loadings** | | |
| --- | --- | --- | --- |
|  | **Baseline** | **Week 8** | **Week 16** |
| CFI | 0.94 | 0.96 | 0.97 |
| RMSEA | 0.127  [0.120; 0.133]_90% CI_ | 0.138  [0.131; 0.145]_90% CI_ | 0.127  [0.120; 0.134]_90% CI_ |
| Hold a plate | 0.77 | 0.86 | 0.90 |
| Pour liquid | 0.81 | 0.88 | 0.90 |
| Unscrew lid | 0.78 | 0.83 | 0.84 |
| Cut food | 0.86 | 0.89 | 0.89 |
| Use fork | 0.85 | 0.86 | 0.86 |
| Prepare food | 0.80 | 0.85 | 0.88 |
| Wash dishes | 0.81 | 0.82 | 0.87 |
| Button clothing | 0.83 | 0.87 | 0.87 |
| Open close zipper | 0.83 | 0.89 | 0.88 |
| Pull on socks | 0.82 | 0.85 | 0.86 |
| Tie shoelaces | 0.83 | 0.89 | 0.87 |
| Hold toothbrush | 0.88 | 0.87 | 0.88 |
| Wash hands | 0.79 | 0.84 | 0.87 |
| Shower or bath | 0.80 | 0.87 | 0.89 |
| Brush hair | 0.85 | 0.92 | 0.88 |
| Put cream on face | 0.81 | 0.83 | 0.84 |
| Write short sentence | 0.81 | 0.87 | 0.84 |
| Type on keyboard | 0.76 | 0.83 | 0.85 |
| Press buttons | 0.80 | 0.82 | 0.86 |
| Press cell phone | 0.77 | 0.82 | 0.85 |
| Open door | N/A | N/A | N/A |
| Turn a key | 0.84 | 0.89 | 0.92 |
| Use scissors | 0.83 | 0.89 | 0.88 |
| Pick up coins | 0.80 | 0.82 | 0.79 |
| Sweep floor | 0.80 | 0.85 | 0.79 |
| Grip steering wheel | N/A | N/A | N/A |

CFI, comparative fit index; CI, confidence interval; HDISS-DU, Hand Disability in Systemic Sclerosis-Digital Ulcers; N/A, not applicable; RMSEA, root mean square error of approximation.

## Supplementary material references

1. Khanna D, Denton CP, Merkel PA, Krieg T, Le Brun FO, Marr A, Papadakis K, Pope J, Matucci-Cerinic M, Furst DE *et al*: **Effect of macitentan on the development of new ischemic digital ulcers in patients with systemic sclerosis: DUAL-1 and DUAL-2 randomized clinical trials**. *JAMA* 2016, **315**(18):1975**-**1988.

2. Farrar JT, Pritchett YL, Robinson M, Prakash A, Chappell A: **The clinical importance of changes in the 0 to 10 numeric rating scale for worst, least, and average pain intensity: analyses of data from clinical trials of duloxetine in pain disorders**. *The journal of pain : official journal of the American Pain Society* 2010, **11**(2):109**-**118.

3. **Guidance for Industry. Patient-reported outcome measures: Use in medical product development to support labeling claims** [<https://www.fda.gov/downloads/drugs/guidances/ucm193282.pdf>]

4. Khanna D, Poiraudeau S, Gelhorn H, Hunsche E, Papadakis K, Perchenet L, Mattera M, Vernon M, Mouthon L: **Development and content validity of the Hand Disability in Systemic Sclerosis-Digital Ulcers (HDISS-DU) scale**. *Arthritis Rheum* 2011, **63**(10 (Suppl)):S726.

5. Steen VD, Medsger TA, Jr.: **The value of the Health Assessment Questionnaire and special patient-generated scales to demonstrate change in systemic sclerosis patients over time**. *Arthritis Rheum* 1997, **40**(11):1984**-**1991.

6. Pope J: **Measures of systemic sclerosis (scleroderma): Health Assessment Questionnaire (HAQ) and Scleroderma HAQ (SHAQ), physician- and patient-rated global assessments, Symptom Burden Index (SBI), University of California, Los Angeles, Scleroderma Clinical Trials Consortium Gastrointestinal Scale (UCLA SCTC GIT) 2.0, Baseline Dyspnea Index (BDI) and Transition Dyspnea Index (TDI) (Mahler's Index), Cambridge Pulmonary Hypertension Outcome Review (CAMPHOR), and Raynaud's Condition Score (RCS)**. *Arthritis Care Res (Hoboken)* 2011, **63 Suppl 11**:S98**-**111.

7. Morrisroe K, Stevens W, Huq M, Sahhar J, Ngian GS, Zochling J, Roddy J, Proudman SM, Nikpour M: **Validity of the Workers Productivity and Activity Impairment Questionnaire: Specific Health Problem (WPAI:SHP) in patients with systemic sclerosis**. *Clin Exp Rheumatol* 2017, **35 Suppl 106**(4):130**-**137.

8. Gentile DA, Woodhouse J, Lynch P, Maier J, McJunkin T: **Reliability and validity of the Global Pain Scale with chronic pain sufferers**. *Pain physician* 2011, **14**(1):61**-**70.
